# Provisioning Vehicular Services and Communications Based on a Bluetooth Sensor Network Deployment

**DOI:** 10.3390/s150612765

**Published:** 2015-05-29

**Authors:** David Perez-Diaz de Cerio, José Luis Valenzuela

**Affiliations:** Universitat Politècnica de Catalunya, C/Esteve Terrades 7, Castelldefels 08860, Spain; E-Mail: valens@tsc.upc.edu

**Keywords:** Bluetooth, vehicular, I2V, R2V, test-bed

## Abstract

It is very common to rule out Bluetooth as a suitable technology for vehicular communications. The reasons behind this decision usually result from misconceptions such as accepting that Bluetooth has a short application range, or assuming its connection setup is not fast enough to allow communication which involves high speed moving nodes. This paper refutes those assertions and proposes the use of Bluetooth not only for Infrastructure-to-Vehicle (I2V) or Road-to-Vehicle (R2V) communications, but also for Vehicle-to-Vehicle (V2V) or Vehicle-to-Infrastructure (V2I) communications. This novel proposal is based on using the remote name request procedure of the standard, combined with an adjustment and optimization of the parameters present in the inquiry and page procedures. The proposed modifications reduce the information exchange delay, thus making Bluetooth a suitable technology for high-speed vehicle communications. The feasibility of the proposed scheme has been validated through experimental tests conducted in different scenarios: laboratory, a real highway and a racing test circuit. There, the communication system was installed in a vehicle circulating at speeds of up to 250 km/h, whereas autonomous devices were disseminated throughout the road path to communicate with the on board devices obtaining satisfying results.

## 1. Introduction

In the last few years, we have witnessed a technological leap in the automotive industry. Hundreds of sensors and information systems have been integrated into vehicles to continuously monitor their parameters. This entails that vehicles now incorporate increasingly complex communications systems to efficiently connect all subsystems.

Including communications systems in vehicles is producing significant advances in the automotive industry. However, in the future it is expected that communications between vehicles and between vehicles and infrastructure will play an important role in the provision of services on board. Safety and mobility applications are likely to be the new wave of services in which communication systems and connectivity inside the vehicle will be key to its implementation [1,2,3]. Moreover, the autonomous vehicles, as the Google driverless car, are projects that involve developing technology for communications systems. Regarding connectivity with external devices, this is a new field of application with a potential large projection in the future [4]. This can be further divided into:

- V2I and I2V (Vehicle to Infrastructure and *vice versa*): Provides access to information such as traffic conditions, Internet access information, download content, communications and emergencies.
- V2R and R2V (Vehicle to Road and *vice versa*): Vehicle connections with elements located at the roadside such as road signs, emergency signaling equipment, payment system, *etc*. and in the reverse direction.
- V2V (Vehicle to vehicle): Connection between vehicles: speed control, emergencies, entertainment.

**Figure 1:**
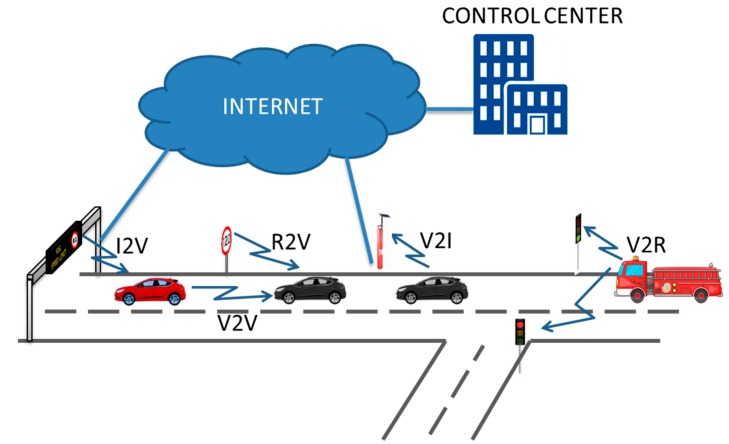
Vehicular network communications.

The difference between V2R/V2I and I2R/I2V [4,5] is subtle and both terms are very often used indistinctly. In fact, the solution we propose can be used for either R2V or I2V communications. As illustrated in Figure 1, the difference resides in whether the exchange of information is limited between the vehicle and the Roadside Unit (RSU) or it has other sources/destinations in the network.

R2V applications can be classified as high-priority and low-priority safety applications. High-priority safety applications are targeted at avoiding imminent crashes and minimizing the damage when collisions become unavoidable. These applications impose several requirements on the communication system. The communication latency has to be minimized in order to offer the driver or the vehicle enough time to take action and the communication system must provide high reliability. Low-priority safety applications aim to increase driver safety, but do not require an immediate driver reaction, because the hazards are not imminent. This includes warning the driver of weather and road conditions, traffic, construction zones, reduced visibility or warning signals.

For example, some of the most important manufacturers of vehicles like Audi, Opel, Ford, Volvo, BMW, Mercedes, Saab and Volkswagen propose mechanisms to read road signs with cameras. Road Sign Information supports the driver by displaying road signs in the instrument display. The forward-looking camera can detect speed limit signs as well as “no overtaking” signs [6]. The road sign icon is shown until another sign is detected. Road Sign Information can be combined with the Speed Alert function, which provides the driver with a visual warning on the speedometer if the speed limit is exceeded. Instead of using these cameras and image processing, a wireless device could read these signals further away and even under unfavorable conditions like heavy rain, fog or other handicaps that could prevent to read visually a sign.

The use of wireless elements for sensing and vehicular communications has faced many challenges. The main cause of the problem has been the lack of standardization, resulting to vendor-defined sensing devices that were often incompatible and hard to integrate to a single practical system. In the last few years, a set of new wireless communication standards and pre-standards with high mobility support, low latency and high data rate has emerged. Some of these technologies include Dedicated Short Range Communications and Wireless Access in Vehicular Environment (DSRC—WAVE) or 802.11p, WiMAX or 802.16, and Mobile Broadband Wireless Access or 802.20, Bluetooth or 802.15 [7]. These wireless technologies can enable a wide range of safety and mobility applications such as video surveillance, voice over Internet Protocol (IP), traffic management, traveler information, wireless internet service or data sensing.

Bluetooth [8] is increasingly used to pair mobile phones to vehicles. Such pairing enables hands-free calling from the vehicle. It also allows a vehicle embedded display unit to be used to control mobile phones, and allows a mobile phone to use the vehicle embedded sound system for reading received messages to avoid driver distractions.

Based on the success of the Bluetooth technology in the field of personal area networks, the main objective of this work is to investigate the feasibility of using Bluetooth-based sensors for V2R/V2I and R2V/I2V scenarios.

Usually these communications are achieved using other technologies and it is possible to find real deployments such as the ones shown in [9,10]. Relevant dissertations can also be found in the literature (e.g., [4]), but they do not discuss the topic in depth. Other works [11,12,13] assure that Bluetooth is only suitable for in-vehicle communications. The reasons behind this very common belief are the connection setup delay [14] or the limited range [15,16,17,18] of the Bluetooth technology. In [19,20] a study of the discovery time of Bluetooth is presented, however all their results are based only on simulations or theory which differ from the obtained with real devices. A more recent study [17], does these tests using real devices, but they do not change the default parameters to optimize the discovery time, except for the interlace mode or the scan window, obtaining discovery times that exceed the second.

In this paper we propose an alternative and new use of the remote name request procedure described in the Bluetooth standard. We assume that the 248 bytes that can be exchanged using this procedure convey enough information to create useful R2V/I2V applications. We also propose to modify, in a way not contemplated in the standard, the parameters present in the inquiry and page procedures to reduce the discovery times. Finally, we measure the range and evaluate the system under these conditions to demonstrate that Bluetooth is a suitable technology for V2R/I2R communications.

In the following section, some of the mechanisms available to transfer data over a Bluetooth connection are discussed. Nevertheless, all of them require a first step to establish a link: the inquiry/page procedures. These procedures and the parameters they depend on are described in Section 2. As of this point, the different experiments and results are presented in Section 3, starting with laboratory tests to determine the best set of parameter values, followed by some coverage tests, speed tests and a real deployment on a highway to demonstrate the feasibility of using Bluetooth in R2V/I2V communications.

## 2. Understanding Bluetooth Connections

Bluetooth operates in the 2400–2483.5 MHz range. This is in the globally unlicensed Industrial, Scientific and Medical 2.4 GHz short-range radio frequency band. The effective range varies due to propagation conditions, the antenna and the receiver sensitivity. Range is also power-class dependent. For instance two Class 1 devices which transmit with an equivalent isotropically radiated power of 100 mW combined both with high sensitivity receivers (−93 dBm) and 3 dBi antennas can theoretically allow ranges up to five kilometers when a free space propagation model [21,22] is considered (Equation (1)):

(1) $$P_{R}=P_{T}\cdot \;G_{T}\cdot G_{R}\cdot {\left(\frac{\lambda }{4\pi d}\right)}^{2}$$

In this equation, *P* is power in Watts, λ is the electromagnetic wavelength, *d* is the far-field line-of-sight distance between the antennas, *G* is the antenna gain and the subscripts *R* and *T* denote the receiver and transmitter, respectively. However, in practice, due to other impairments, e.g., the presence of obstacles or the effect of the ground, weather conditions, the losses introduced by both the antenna cable and the connectors, *etc.*, the range is limited to about two kilometers. This range can be achieved in low visibility conditions such as rain or fog. In addition, radio signals can avoid obstacles such as other autos, trucks, *etc.* This mobility scenario raises some serious questions about the amount of time that Bluetooth takes to establish a connection. If this process takes too long, then either no connection can be made or there will not be enough time to do any useful data transfer. This work demonstrates that discovery and connection time can be improved using an appropriate connection setup.

### 2.1. Data Transfer Mechanisms in Bluetooth

**Figure 2:**
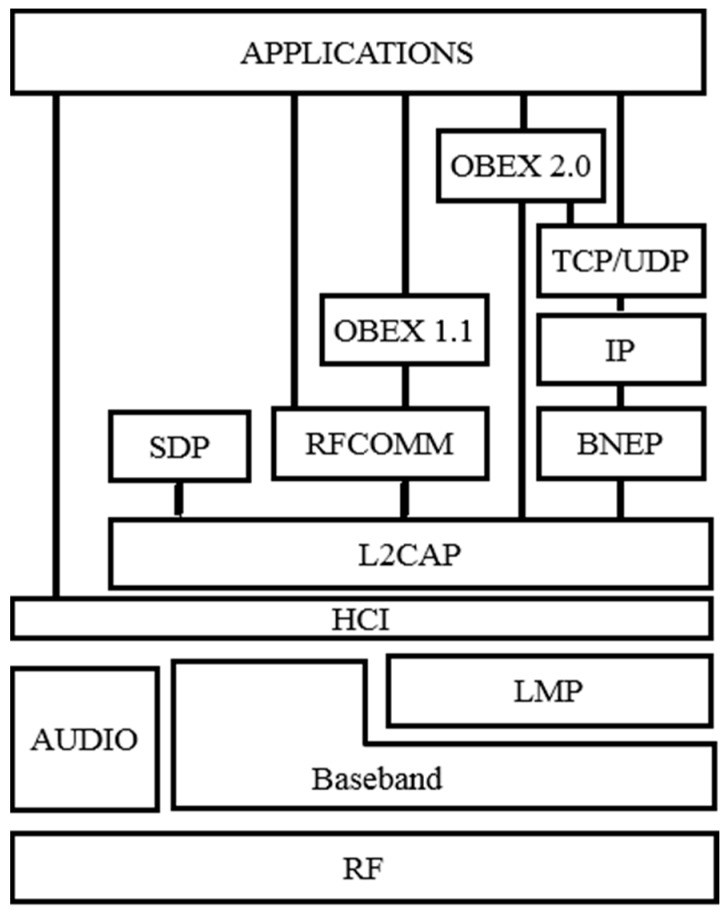
Bluetooth stack protocol.

One of the parameters under evaluation is the time needed to discover and establish a Bluetooth connection and, thus, be in a position to transmit data. The Bluetooth standard offers several mechanisms to enable data transfers, such as establishing a Radio Frequency Communication (RFCOMM) connection, an Object Exchange (OBEX) exchange or even a complete *ad-h*oc network using the personal area network profile. Each one of these modes implies the use of a different set of the protocols from the Bluetooth protocol stack as illustrated in Figure 2. Therefore, each one of them offers different benefits, but none of them is suitable for the intended scenario.

The Link Manager Protocol (LMP) controls and negotiates the operation of the Bluetooth connection between two devices. This includes the set-up and control of logical transports, logical links, and physical links. The LMP supports the remote name request procedure, which enables the acquisition of the name of another device. The name parameter is a user-friendly name associated with the device and consists of a maximum of 248 bytes coded according to the 8-bit Unicode Transformation Format (UTF-8) standard.

**Figure 3:**
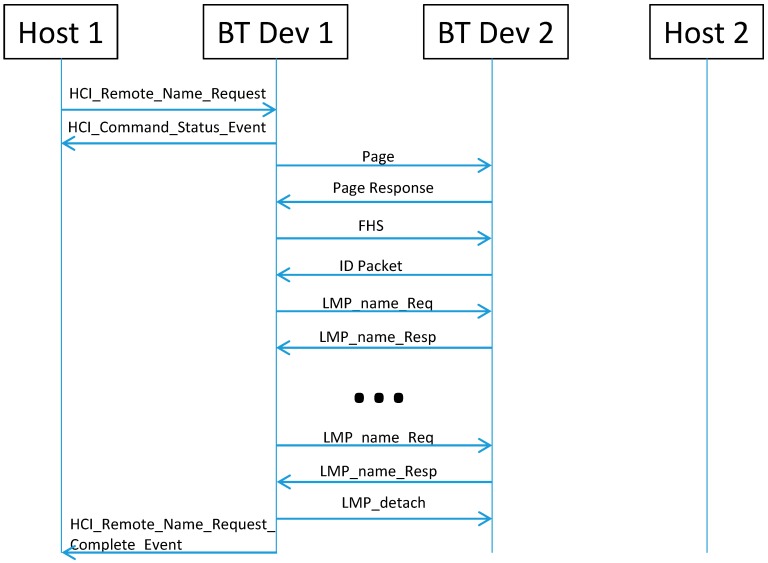
Remote Name Request procedure.

The name parameter can be changed with the Host Controller Interface (HCI) “write local name” command [8]. The HCI implements the HCI commands for the Bluetooth hardware by accessing baseband commands, link manager commands, hardware status registers, control registers and event registers. The HCI “remote name request” command is used to get the name of the remote device without establishing an Asynchronous Connection-Less (ACL) connection. As depicted in Figure 3, the device first establishes a connection with the paging procedure, and then uses the LMP “Name Request”. The “LMP_name_request” and “LMP_name_response” messages can be repeated several times depending on the name length. Finally, it disconnects and returns the name of the remote device back to the host. In this way, the remote name request seems one of the simpler methods to exchange a small data sequence and, therefore, is proposed along the paper as a possible solution for the intended scenario.

The HCI commands are part of the Bluetooth stack that is a generic software stack that includes HCI driver, Logical Link control and Adaptation Protocol (L2CAP), RFCOMM, Bluetooth Network Encapsulation Protocol (BNEP) and Service Discovery Protocol (SDP). This software can be implemented on computers or in embedded systems. As described later, during the tests, a modified stack protocol was used in order to access and change some of the standard configuration parameters of the devices.

### 2.2. Inquiry and Page Mechanisms

All the techniques described in the previous section require establishing first a link between the nodes. This link is achieved after an inquiry/page process and, in order to minimize the connection time, it is necessary to know the tweakable parameters of this procedure.

**Figure 4:**
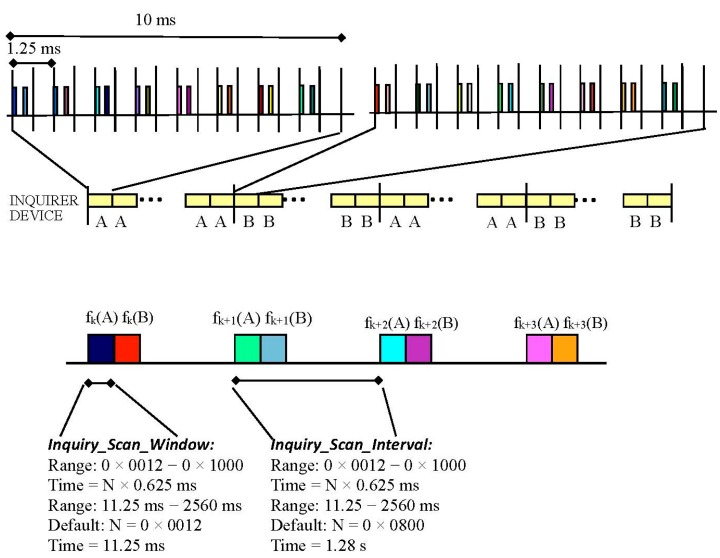
Inquiry process (interlaced).

The inquiry process, illustrated in Figure 4, is used to determine the in-range devices. A device in inquiry state broadcasts IDentity (ID) packets at twice the normal frequency-hopping rate, *i.e.*, it sends two ID packets every 625 μs and then listens for responses the following 625 μs.

An ID packet contains the Inquiry Access Code (IAC). There are two kinds of Inquiry Access Codes, the *General* IAC (GIAC) and the *Dedicated* IAC (DIAC). The GIAC is used in a general case when a device wishes to know all devices in range. The DIAC is more selective and it is used when a device only wishes to know about devices with special characteristics. It is possible to use the DIAC in the target scenario to limit the responses to just a selected number of devices, thus, accelerating the link establishment.

The duration of the inquiry window is controlled by the Inquiry_scan_window (*T*_w_inquiry_) parameter. However, the device can also be set to exit the inquiry state after a certain number of devices (*N*_inq_responses_) have been found. When the inquiry message is received in the inquiry scan substate, the device returns a Frequency Hop Synchronization (FHS) packet containing the Bluetooth device address (BD_ADDR) and other parameters.

The inquiry procedure uses 32 dedicated hop frequencies according to the inquiry hopping sequence. These frequencies are determined by the general inquiry address. The 32 frequencies are divided into two trains, *A* and *B*, each train is 10 ms in length and according to the specification a train must be repeated at least 256 times before a new train is used.

Two types of scans are defined: standard and interlaced. In the case of a standard scan the length of this scan period is denoted T_w_inquiry_scan_ (inquiry_scan_interval). The standard scan is performed at a single frequency. A different channel frequency is selected every 1.28 s. The interlaced scan, depicted in Figure 4, is performed as two consecutive scans of T_w_inquiry_scan_ milliseconds where the first scan is on the normal hop frequency (from train set A or B) and the second scan is from the complementary train set (B or A). If the scan interval is not at least twice the scan window then, interlaced scan shall not be used.

The page mechanism is very similar to the inquiry mechanism. This mechanism is used when the device that initiates the connection operation (paging device) already knows the Bluetooth address of the other device. Thus, it is mandatory to have previously gone through an inquiry process. The paging device sends ID packets whose access code has been generated from the Bluetooth Address of the paged device. In the same way as explained in the inquiry process, from the total of 79 available frequencies, the paging device only uses a subset of 32 frequencies. Then, the first 16 frequencies are transmitted during 10 ms with a frequency hopping of 32 hops per second. These frequencies are repeated N_page_ times. Following, the subset of the remaining 16 frequencies is used. The N_page_ value depends on another parameter, the Scan Repetition mode, as well as the manufacturer implementation.

**Figure 5:**
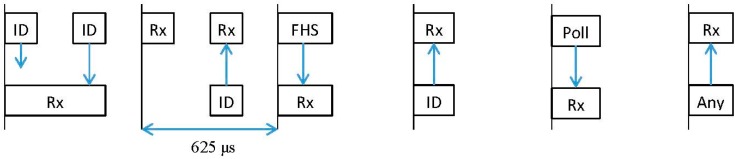
Page process.

When the frequencies between the transmitted packet and the scanning procedure coincide, the receiver returns the ID to the paging device a slot later. After that, a packet interchange is executed to inform of the frequency hopping sequence used and the link is finally established. This process is depicted in Figure 5.

## 3. Evaluating Bluetooth in R2V/I2V Scenarios

Several tests have been conducted to evaluate and demonstrate the feasibility of using Bluetooth in R2V/I2V communications. In this section, the used equipment and the different evaluated scenarios are described.

First, a series of laboratory tests were made with two objectives: to study the most suitable operating parameters of the Bluetooth technology and to determine the best mechanism for sending data using Bluetooth in vehicular environments. In these tests the influence of the inquiry and page parameters over the delay in the device discovery procedures and the transmission of information, the level of signal reception depending on the distance, and the global throughput were evaluated.

The following tests aimed to determine the maximum connection distance between two Bluetooth devices. This coverage is analysed using the received signal strength indicator (RSSI) level as a function of the distance during the device discovery process.

Finally, it was also necessary to evaluate the performance in scenarios where one of the nodes is moving at high speeds in order to demystify the common idea that is not possible to establish a link and transmit data using Bluetooth in vehicular environments. Thus, two different field tests were designed: one at the *Circuit de Catalunya* raceway achieving speeds of up to 250 km/h and the other one on a straight section of a public highway.

### 3.1. Bluetooth Parameters Evaluation

The response time depends on the configuration of the device. Specifically the inquiry/page scan window, the inquiry/page scan interval parameters and the interlaced scan mode have a significant impact on the performance. The definition of these three parameters was explained in Section 3 and depicted in Figure 4.

For these experiments, two laptops with a Bluetooth 2.1 Universal Serial Bus (USB) dongle were deployed maintaining a distance of 2.5 m between them. Both dongles were connected to 3 dBi omnidirectional antennas. The operating system of the computers was linux. Using the utilities included in the BlueZ package the influence of inquiry_scan_window, the interlace mode and inquiry_scan_interval parameters were evaluated.

From the preliminary tests made, we can conclude that increasing the scan window did not produce any benefits so, the minimum value of 18 slots (which is also the default configuration) has been selected for the remaining test. In a similar way, considerably better results were obtained using interlaced scan, so the non-interlaced mode was also discarded.

**Figure 6:**
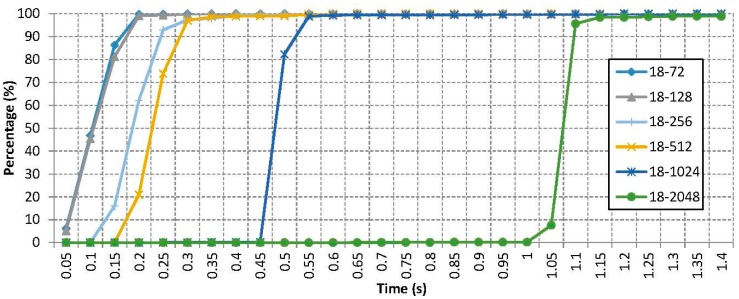
Cumulative probability for different values of the scan interval parameter.

However, some benefits can be found by reducing the scan interval under the default value of 2048 slots. The results are shown in Figure 6. This figure represents the cumulative probability *vs.* the time until an inquiry response is received for different values of the scan interval, for one thousand samples.

The theoretical expected values suggested that this cumulative percentage should follow a linear increment between zero and a certain value that increases with the scan interval parameter. This fact was validated by means of simulations of the inquiry procedure with two devices involved. In these simulations, the first device was configured to be in inquiry scan mode and the other one initiated the inquiry discovery procedure (t_0_) after a random time period. Both devices employed different frequency hopping sequences taken from the Bluetooth standard [8]. The process was considered completed (t_f_) when the frequencies of both devices coincide. The statistics of the discovery time (t_f_ − t_0_) were extracted after five thousand realizations which corroborate the theory.

The obtained results in practice do not follow exactly this behaviour because the device driver is not fast enough to communicate instantaneously when the inquiry is complete. However, the results allow determining the time lapses where an inquiry request is completed with total probability. As can be seen, reducing the scan interval improves considerably the time to find a device keeping it under half a second.

**Figure 7:**
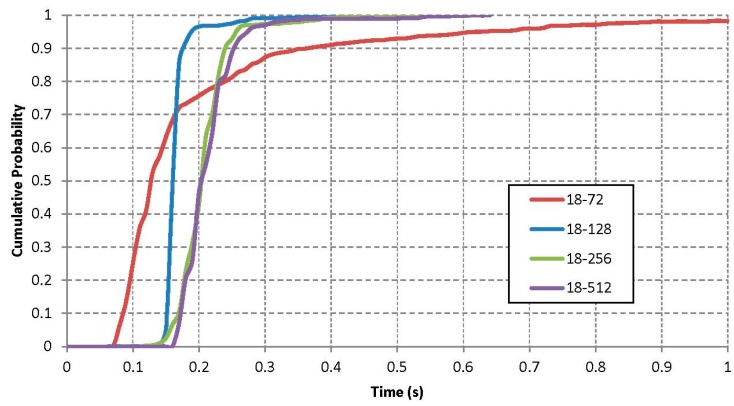
Remote Name Request time response.

In order to complete a data transmission, apart from doing an inquiry, it is necessary to establish a full link. This requires one to follow both an inquiry and a page procedure. These two processes can be done separately or in a block to accelerate the connection. As explained in Section 2.1, one of the shortest data interchange mechanisms is the remote name request, which allows to transfer up to 248 bytes of data from one device to another. Figure 7 depicts the results of doing an inquiry + page process and a remote name request with different combinations of scan interval and an 18 slots scan window, in order to compare all the results.

As can be observed, the extra time of doing a full remote name request procedure just after an inquiry is almost negligible and a transmission of up to 248 bytes of data can be achieved in less than 200 ms. In this figure, the combination 18/72 shows a peculiar behaviour, since it needs more time than expected. Theoretically, the 72 slots are sufficient to fulfil the need for 18 + 18 slots of the inquiry process and the 18 + 18 slots of the page process. However, the hardware is not able to commute fast enough between the inquiry and page mechanisms. Apart from this anomaly, the conclusions extracted are similar: reducing the scan interval improves considerably the time to connect and transmit the data. Nevertheless, with a smaller scan interval, there is less time left for other transmissions and it will have a negative impact when the amount of data to be transmitted increases. For example, Table 1 shows this effect when a variable size packet is transmitted using OBEX. It is necessary to take into account that the values shown on the table only include the average time of the data transfer. The time to establish a link and the start-up of the OBEX protocol are not reflected.

Thus, looking at the time needed to transmit a 250 bytes data packet to be equatable with the size used in the remote name request, as expected, using OBEX requires at least the double of time than the remote name request. Establishing a network using the personal area network profile was also evaluated. Nevertheless, this process requires not only establishing a link but also a full IP network including the request of an IP address. This makes that the establishment time is extremely high for the proposed application.

**Table 1:** Time (s) to transmit a data packet using OBEX (scan window/scan_interval).

Data Bytes	18/128	18/256	18/252	18/1024	18/2048
1	0.55	0.35	0.35	0.29	0.25
10	0.55	0.36	0.35	0.29	0.27
100	0.60	0.42	0.36	0.30	0.30
1000	0.61	0.43	0.37	0.34	0.31
10,000	1.51	1.03	0.69	0.67	0.64
100,000	10.89	6.97	5.10	4.74	4.40
1,000,000	134.83	62.92	43.84	38.02	36.36

In this way, the remote name request procedure was the method finally employed during the experimentation. At the inquiry process held before the remote name request, a DIAC (explained in Section 2.2) and an 18/128 Inquiry/page scan window/scan interval relation in interlaced mode were used to optimize the results obtained.

### 3.2. Field Tests

In the light of the obtained results, the next step was to evaluate the performance of the I2V/R2V communication using Bluetooth in a controlled environment, trying to keep always the same conditions, but, in any way, very similar to the real scenario found in a highway. First, it was necessary to do some coverage tests to assure the viability in a vehicular environment with a high speed mobile node.

#### 3.2.1. Coverage Tests

The coverage tests were held in several scenarios where the differences in the environment, the interfering elements and the propagation impairments are reflected in the measurements.

**Figure 8:**
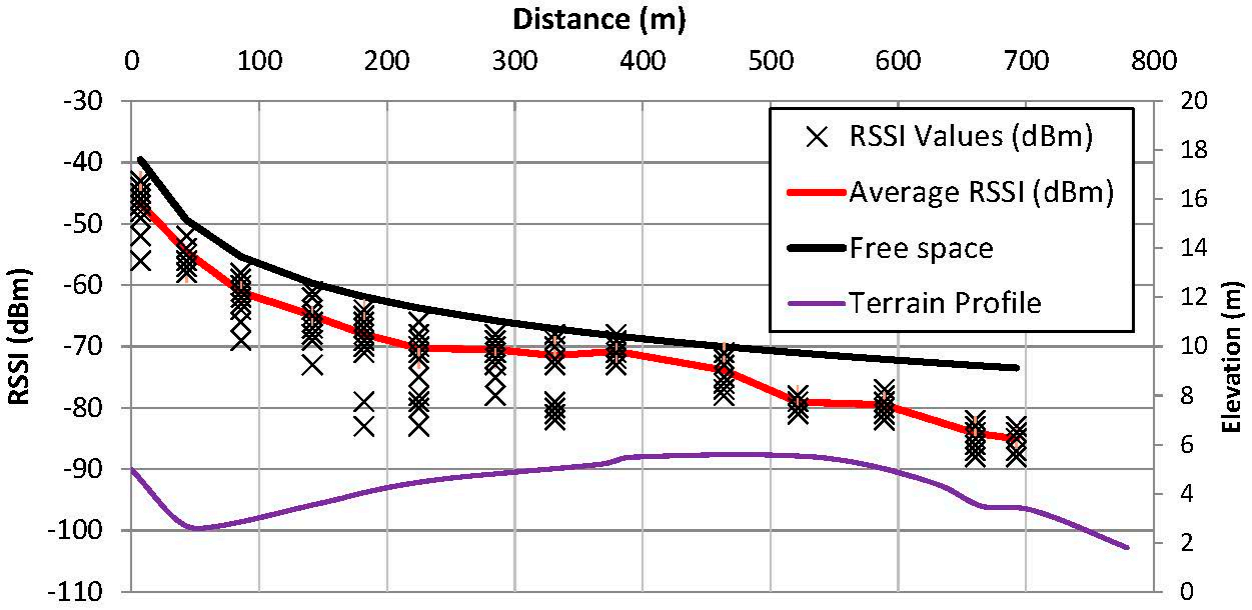
RSSI at the campus.

The first test was done in the surroundings of the campus; see Figure 8. There was one fixed transmitter placed at one point and a mobile receiver moving away from that point. Both devices used a commercial, unmodified, Bluetooth dongle of Class 1 (100 mW), specifically, the Parani UD100 model from Sena Technologies Inc. (San Jose, CA, USA).

Figure 8 reflects the values of the Received Signal Strength Indicator (RSSI) of the captured packets. The lowest line shows the orographic profile of the followed path, with the height in meters over the sea level represented at the right axis of the figure. The X markers depict the RSSI value in dBm of each received packet and the red line shows the average of this value. The measured sensitivity of the receiver was around −86 dBm and as can be seen this allowed covering a range of near 700 m. The theoretical received power using a free space propagation model (Equation (1)) is included depicted in black as a reference, and it shows clearly the same trend than the empirically obtained values.

Additionally, another similar test was done in the seafront of Castelldefels. Due to the better propagation conditions (absence of vegetation and other interfering objects), remote name request packets could be received at a distance of up to 950 m.

#### 3.2.2. Circuit the Catalunya

The aim of this experiment was to test the coverage, the response time and the maximum speed at which the mobile could establish a communication and a short data transmission. For this, a Bluetooth device was placed at the top of an eight meter infrastructure located at the end of the main straight of the Circuit de Catalunya (Montmeló speed circuit). The mobile device was placed inside a racing car (Ferrari F430) with a driver and one operator. This device was controlled by a laptop with an attached a global positioning system (GPS) to obtain the time, position and speed data for each of the events happening during the data transmission.

**Figure 9:**
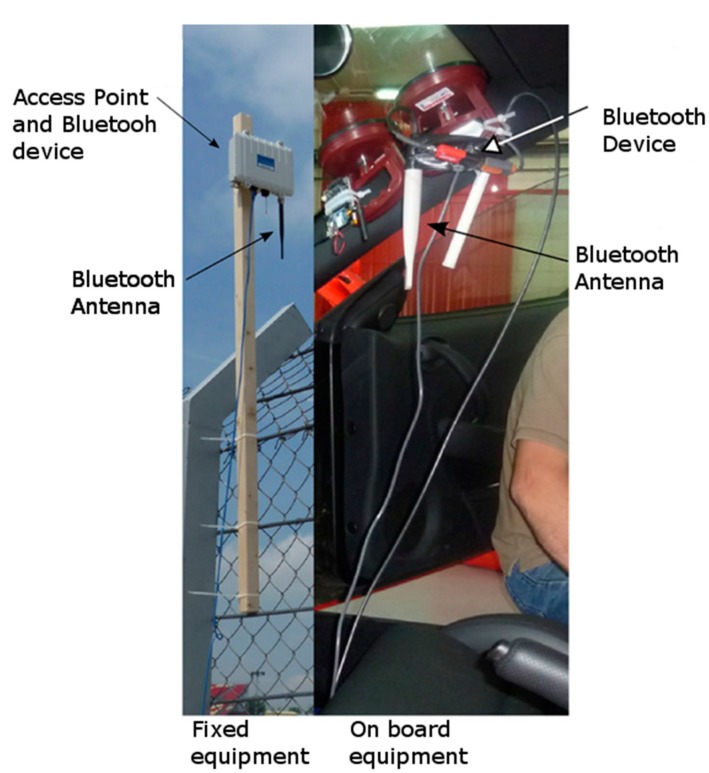
Test equipment.

**Figure 10:**
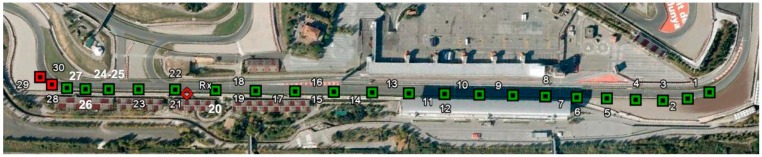
Remote name requests received at the Circuit the Catalunya speed circuit.

As can be observed in Figure 9, the factory antennas of the Sena devices were substituted by two 3 dBi omnidirectional antennas to counteract part of the effect of placing the device inside the vehicle. The distance between the first line-of-sight point and the fixed device is approximately one kilometre. The vehicle leaves the last turn at 160 km/h (Figure 10 point #1) and reaches a top speed of near 250 km/h near the fixed Bluetooth device (red diamond near point #21).

**Figure 11:**
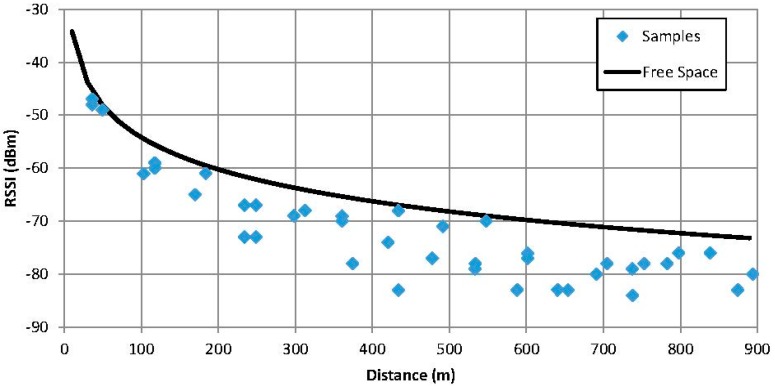
RSSI of the received remote name answers.

The results shown in Figure 11 represent the RSSI of the successful received answers to the remote name requests. The upper line marks the orographic profile and the lower line the theoretical results for the free space propagation model (Equation (1)). As it can be observed, the real results follow the same trend than the theoretical values.

**Figure 12:**
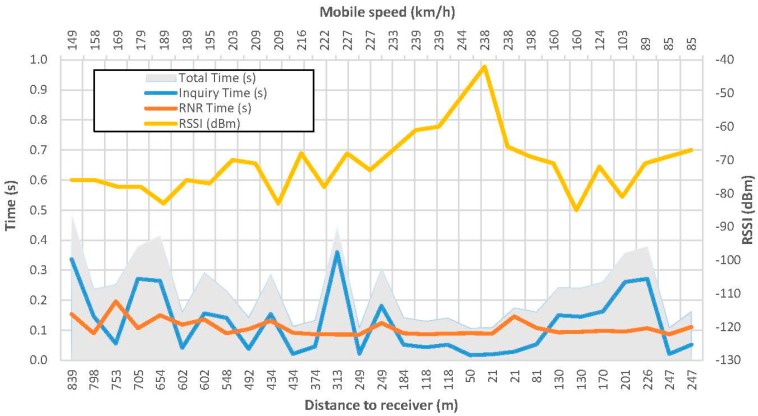
Results for a selected single pass.

A more complete example can be seen in Figure 12. These results are obtained for a single pass, where up to 30 remote name request answers were received. These points correspond with the ones shown in Figure 10.

Figure 12 depicts the speed of the mobile node in each point in the upper axis, the distance to the receiver in the lower axis, the inquiry process and remote name request time in the left axis and the received RSSI in the right axis.

As it can be observed, the vehicle starts to brake just at the point where the fixed equipment was placed. Since the on board equipment was placed in the front windscreen, the RSSI decreases faster once surpassed the fixed measuring point. Paying attention to the connection times, the average connection time is 230 ms, distributed almost equally between the inquiry process and the remote name request procedure.

#### 3.2.3. Highway

**Figure 13:**
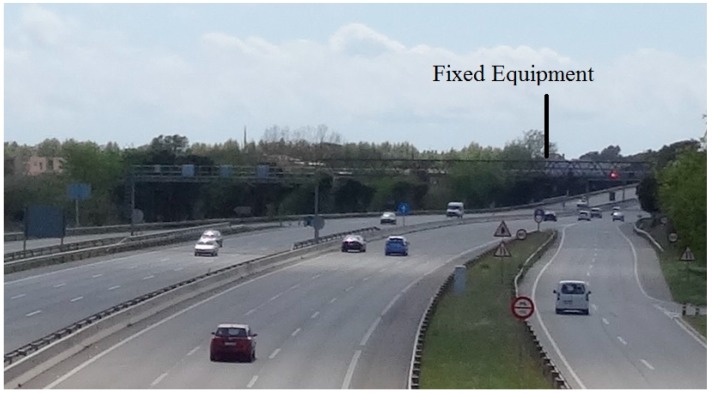
Deployment on a real highway.

In a real highway environment, in addition to being at high speed, there can be several vehicles (and potentially several types, e.g., cars, trucks, high-profile vehicles) surrounding the target vehicle and potentially causing interference. So, in this last experiment, the system was tested over a more realistic and less controlled environment. The same equipment and configuration used at the speed circuit was employed. However the fixed equipment was placed around 10–15 m over the road which could be equivalent to the height of a lamppost or a traffic light. The location of the fixed equipment is marked in Figure 13.

**Figure 14:**
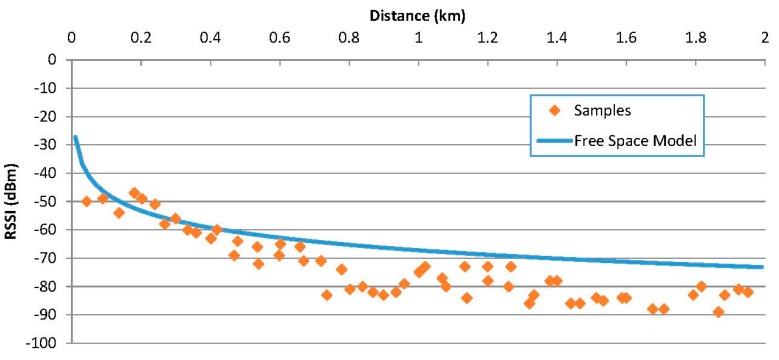
Received RSSI in a real highway.

Regarding connection times, *i.e.*, the addition of the time spent in the inquiry procedure and the remote name request, are similar to the obtained in the speed circuit, around 200 ms. The main difference observed when comparing Figure 11 and Figure 14 is the increase of the coverage. With this configuration, it is possible to receive a remote name request up to two kilometres away from the fixed equipment. This enhancement is due to the higher position of the fixed equipment which improves the propagation conditions. If the fixed equipment is placed at the height of a road sign (2–3 m) the range is reduced to around 700 m.

## 4. Conclusions

Several real experiments have been designed, conducted, and discussed in this paper to demonstrate that in R2V/I2V communications it is possible to use Bluetooth, which is usually pushed into the background and its use is reduced to only intra-vehicular communications. The presented proposal refutes the reasons behind this decision which generally correspond to prejudices like accepting that Bluetooth has a short application range, or assuming its connection establishments are not fast enough to allow a communication which involves a high speed moving node.

One of the hypotheses assumed is that a 250 bytes data packet can convey enough information to make useful R2V/I2V applications and, thus, the use of the remote name request procedure is proposed against other connection mechanisms. Using this procedure and optimizing the parameters of the standard inquiry and page processes, a message of up to 248 bytes can be transmitted in average under less than 200 ms, even when the mobiles nodes are travelling at high speeds (<250 km/h).

On the other side, it has been demonstrated that this data exchange can be completed within wide coverage areas, up to two kilometres, with standard and commercial devices equipped with omnidirectional antennas. All these results are obtained by means of real deployments, so under these conditions we can conclude that Bluetooth is a suitable technology that should be taken into account when designing sensors for complex vehicular communications.
